# Malignant Phyllodes Tumor in an Adolescent Female: A Rare Case Report and Review of the Literature

**DOI:** 10.1155/2020/1989452

**Published:** 2020-02-28

**Authors:** Gabriel S. Makar, Michael Makar, Joanna Ghobrial, Kathryn Bush, Ryan Allen Gruner, Thomas Holdbrook

**Affiliations:** ^1^Cooper Medical School of Rowan University, 401 Broadway Ave, Camden, NJ 08103, USA; ^2^Robert Wood Johnson University Hospital, 1 Robert Wood Johnson Pl, New Brunswick, NJ 08901, USA; ^3^AT Still University School of Osteopathic Medicine, 5850 E Still Cir, Mesa, AZ 85206, USA; ^4^Cooper University Hospital, Department of Surgery, 1 Cooper Plaza, Camden, NJ 08103, USA; ^5^Cooper University Hospital, Department of Pathology, 1 Cooper Plaza, Camden, NJ 08103, USA

## Abstract

Primary breast neoplasms are rare in adolescent females, most of which are benign. Phyllodes tumors constitute a remarkably small subset of breast neoplasms (0.3-0.9%) with malignant phyllodes tumors being even more uncommon. Malignant phyllodes tumors tend to progress rapidly though only 1.5% metastasize. They are also associated with a higher rate of recurrence than their benign counterparts, underlying the importance of adequate surgical margins. It is therefore imperative to be able to identify these tumors early allowing for prompt resection and close follow-up. Here, we present the rare case of a 17-year-old female presenting with a rapidly enlarging breast mass, which was ultimately found to be a malignant phyllodes tumor. We further performed a review of the literature to highlight only 22 other cases reported in adolescent females.

## 1. Introduction

Primary breast neoplasms are extremely rare in adolescent females and most are benign in nature. The incidence of primary breast tumors in females under the age of 20 is approximately 1 in one million [1]. Phyllodes tumors of the breast, previously known as cystosarcoma phyllodes, are rare fibroepithelial tumors that constitute approximately 0.3–0.5% of all breast neoplasms [2, 3]. The incidence for these tumors is remarkably low with an estimated 2.1 per one million women with a higher prevalence among Latina whites [4]. They occur most commonly during the late fifth decade of life in females and are even more rarely do they occur in men [5, 6]. Clinically, these tumors present as palpable, sometimes rapidly enlarging masses of the breast with a median size of approximately 4 cm [7]. Additionally, malignant phyllodes tumors are, on average, larger than their borderline or benign counterparts [6].

Malignant phyllodes tumors account for approximately 6.5-27% of all phyllodes tumors [5, 8–11]. Compared to benign and borderline phyllodes tumors, malignant tumors contain a higher rate of disease recurrence, decreased overall survival, and distant metastasis [5, 10]. Although they are more aggressive, cause-specific survival for these patients was 91%, 89%, and 89%, at 5, 10, and 15 years, respectively [12]. The median age of diagnosis remains similar to benign phyllodes at around 50 years old [12].

Herein, we perform a review of the literature and present the rare case of an adolescent female with a malignant phyllodes tumor.

## 2. Case Description

**Figure 1:**
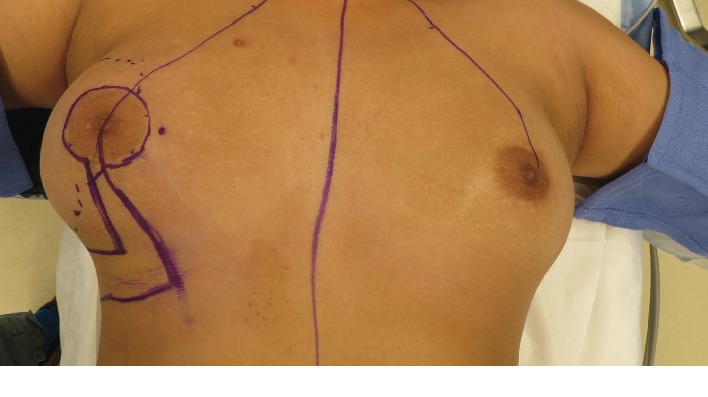
Preoperative image capturing prominence of tumor size and breast distortion relative to contralateral breast.

A 17-year-old female presented to the emergency department (ED) regarding an enlarging right breast mass. She noticed the mass 1 week prior to presentation. It was associated with mild intermittent breast pain and severe periodic episodes of pain rated 10/10. No relieving or exacerbating factors were noted. The patient denied any redness or thickening, nipple discharge, nipple inversion, and any noticeable changes on the contralateral breast. Her breast sizes had not changed relative to one another. She had no previous history of breast biopsies or abnormal breast imaging. She denied additional symptoms including bone pain, rib pain, headache, and shortness of breath and was otherwise in her usual state of health. Her family history was significant only for a maternal aunt with ovarian cancer. The patient underwent menarche at the age of 12. There was no history of use of exogenous hormones to date. Her last menstrual period was approximately 25 days prior to presentation to the ED. Physical exam revealed a large, nontender approximately 15 × 11 cm mass occupying the majority of her right lateral breast (Figure 1). The left breast did not appear to have any masses, inflammatory skin changes, or nipple-areolar complex abnormalities. There was no axillary, infraclavicular, or supraclavicular lymphadenopathy appreciated on exam.

**Figure 2:**
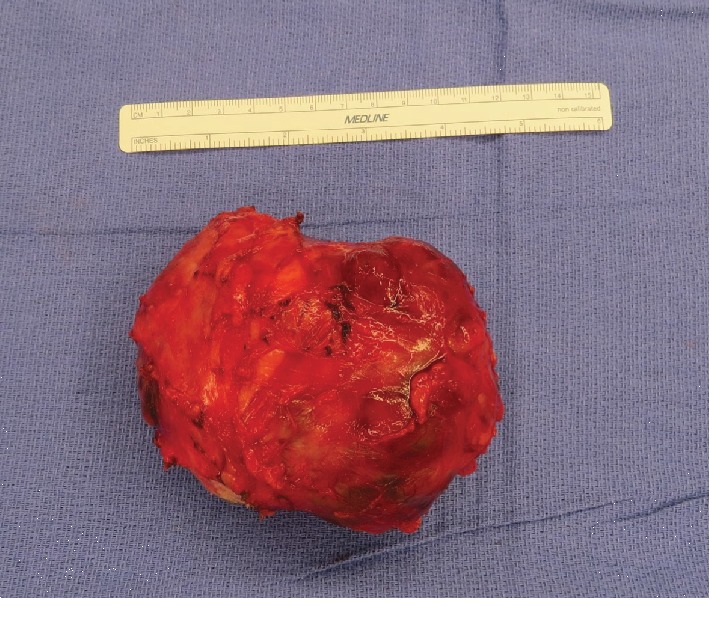
Intraoperative image of excised tumor measuring 12.5 cm.

**Figure 3:**
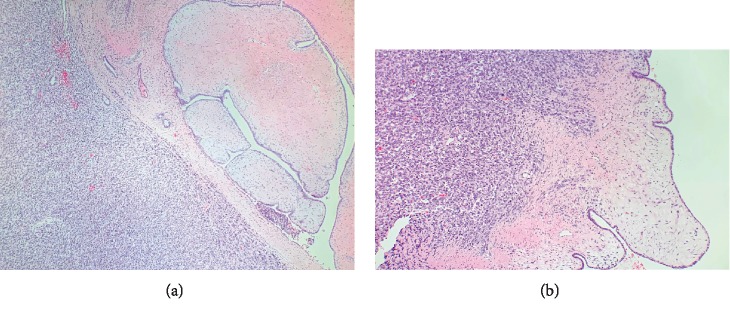
Malignant phyllodes tumor. (a, b) The characteristic leaf-like architecture with epithelium lined clefts is present (right) next to prominent hypercellular stroma (left) (H&E, ×40 (a), ×100 (b)).

**Figure 4:**
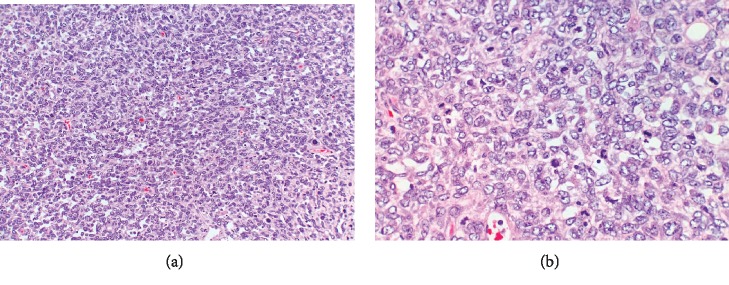
Malignant phyllodes tumor. (a, b) There is marked nuclear pleomorphism and mitotic activity (H&E, ×200 (a), ×400 (b)).

**Figure 5:**
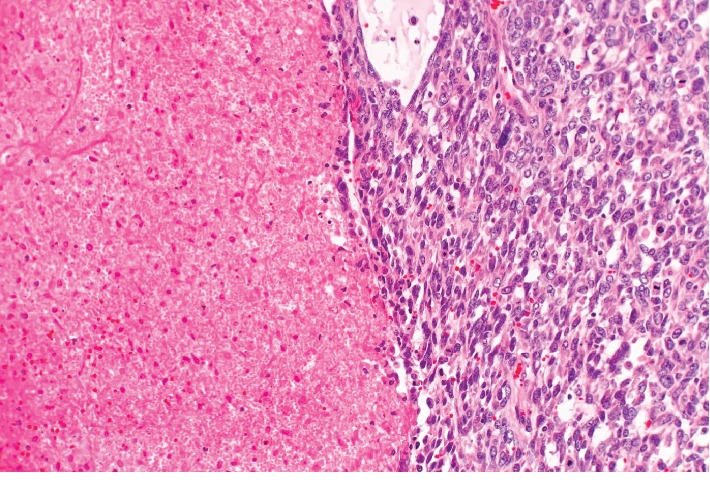
Malignant phyllodes tumor. An area of tumor cell necrosis (left) (H&E, ×200).

Breast ultrasound at the time of presentation revealed a 11.0 × 7.2 × 5.1 cm complex heterogeneous hypoechoic solid mass, which demonstrated mild internal vascularity on color Doppler flow. The mass was noncompressible with surrounding peripheral fluid. The patient underwent an ultrasound-guided core needle biopsy of the mass, which revealed a fibroepithelial tumor. Final pathology favored phyllodes tumor. The patient was also seen by plastic surgery regarding the anticipated large defect post resection of the tumor. Given the pathological findings, the patient underwent a wide right breast mass excision with a complex closure of the 10 cm wound. Pathologic examination revealed a 12.5 cm malignant phyllodes tumor (Figure 2). Histologic sections of the tumor showed the characteristic leaf-like architecture of phyllodes tumor with areas of prominent hypercellular stroma. The hypercellular stroma displayed marked nuclear pleomorphism and mitotic activity as well as areas of necrosis (Figure 3–5). The tumor was within 0.5 mm of the closest resection margin. Therefore, per the 2019 National Comprehensive Cancer Network guidelines, the patient was sent back to the operating room for reexcision lumpectomy to ensure full 1 cm margins around the malignant tumor. A lateral intercostal artery perforator flap was performed for appropriate wound coverage.

The patient has been doing well since the time of surgery with no current signs of metastasis or local recurrence. Chemotherapy was not currently indicated, and although radiation therapy was recommended, the patient refused radiation and opted for close observation [13]. Workup for metastatic and recurrent disease has been negative to date.

We additionally performed a literature review of adolescent females, defined as those 18 years of age or younger, with a confirmed diagnosis of a malignant phyllodes tumor (Table 1). Our report relies predominantly on the review attained from Levêque et al. who reviewed 18 adolescent females with malignant phyllodes tumors and presented a patient of their own (total 19 patients) [14–29]. Our review includes three additional patients for a total of 22 affected adolescent females [30, 31].

## 3. Discussion

Primary breast neoplasms are uncommon among adolescent females and may be misdiagnosed as a physiologic breast mass, as these are more common in this demographic. Phyllodes tumors constitute a rare subset of breast neoplasms (<1%), with a predilection for women in their late fifth to sixth decade of life and malignant phyllodes tumors account for an even smaller cohort (6.5–27% of all phyllodes tumors). Moreover, malignant phyllodes tumors occur in a population comparable to benign tumors, with rare occurrences in the adolescent population. Our report adds to the scarce literature of adolescent females with malignant phyllodes tumors and provides an overview of these tumors in the adolescent female population.

Phyllodes tumors are fibroepithelial neoplasms of the breast, which can histologically resemble fibroadenomas but typically display distinct stromal hypercellularity, prominent intracanalicular growth pattern, and characteristic leaf-like architecture. The World Health Organization (WHO) classifies phyllodes tumors into benign, borderline, and malignant categories based on the degree of stromal hypercellularity, cytological atypia, mitotic activity appearance of tumor border, and stromal overgrowth [32, 33].  Table 2 contains the current WHO criteria for diagnosis of phyllodes tumors [34]. The intracanalicular growth pattern along with hypercellularity is essential in differentiating fibroadenomas from benign phyllodes tumors, which can at times be difficult. Additionally, progression of fibroadenoma to malignant phyllodes tumors is not unknown as seen in case number 20 (Table 1). In a review of 36 malignant phyllodes tumors, 11 (30.6%) were given a diagnosis of primary fibroadenomas and experienced recurrence as malignant phyllodes tumors [35]. To properly capture these tumors, the growth rate must be observed closely. Studies suggest growth more than 20% in 6 months may be concerning for phyllodes tumor over fibroadenoma and constitutes surgical excision or biopsy [36, 37].

Formulating a proper diagnosis for patients presenting with breast masses can be stratified based on the patient's age. Less than 10% of phyllodes tumors occur in females younger than 20 years of age [15]. With malignant tumors also being tremendously rare in this population, there is a high likelihood of missing such tumors. These tumors can also be aggressive and rapidly progressive as seen in our case and previous reports [11, 15]. Ample detection of these tumors requires prompt biopsy, as was done in our case, to rule out such aggressive malignancies. After the diagnosis is made, appropriate negative margins must be established to prevent tumor recurrence. For patients with malignant phyllodes tumor, consensus remains that negative margins of 1 to 2 cm be obtained for optimal excision or simple mastectomy if unable to reach such margins [38].

Recurrence rates for benign, borderline, and malignant tumors vary vastly among studies. The literature, in concordance with the work done by the WHO, suggests that recurrence among benign, borderline, and malignant phyllodes tumors is 10–17%, 14–25%, and 23–30%, respectively [32]. Benign phyllodes tumors may be followed closely even with positive margins; however, malignant tumors must have confirmed negative margins given the high rate of recurrence and even death (22%) [32, 39]. Adolescents are said to have a higher rate of recurrence compared to their adult counterparts and therefore should be observed closely with routine follow-up even in light of negative margins [21]. Studies suggest Asian women appear to be at a higher risk of recurrence than non-Asian's [40]. However, a study utilizing the Surveillance, Epidemiology, and End Results Program (SEER) database found that there was no difference in survival among white, black, and “other” ethnicities [41]. Further research is necessary to identify the true prevalence and incidence of disease among other ethnicities and in younger populations.

## 4. Conclusion

Given the unique and uncommon nature of primary breast tumors in adolescent females, appropriate assessment and management must be achieved to prevent misdiagnosing rare, aggressive tumors. It is necessary to be vigilant in the diagnosis and prompt management of rare tumors such as phyllodes tumors, due to the possibility of malignancy and rapid growth. Key features, such as large and rapidly growing mass, can aid in this diagnosis, but biopsy is necessary to characterize the suspect mass. We report the rare case of a rapidly growing malignant phyllodes tumor in an adolescent female.

## Figures and Tables

**Table 1:** Literature review of malignant phyllodes tumors in adolescent females (part 1).

Case number	Age (years)	Delay (months)	Size (cm)	Treatment	Evolution	Result	Histology	Author
1	16	Short	9 × 8 × 7	Tumorectomy	2 recurrences at 12 months, 18 months, subcutaneous mastectomy+reconstruction	GH at 48 months	NS	Adami et al.
2	14	1.5	7 NS	Tumorectomy	—	GH at 48 months	NS	Azzopardi
3	15	?	8 × 8 NS	Tumorectomy, radiotherapy	2 pregnancies and 2 normal deliveries	GH at 66 months	NS	Rissanen and Holsti
4	17	14	9 right	Mastectomy	—	GH at 35 months	Lipomyxosarcoma	Mollitt et al.
5	16	0.5	7.5 NS	Tumorectomy	2 recurrences at 5 months, 9 months	GH at 37 months	Clear outlines with repression, mitoses 51/10 HPF, atypical aspect 3+, stroma fibrolipochondro sarcoma	Pietruszka and Barnes
6	17	NS	2.5-5 right	Tumorectomy	Adenofibroma at 8 months, tumorectomy	GH at 30 months	NS	Contarini et al.
7	12	6	13 NS	Mastectomy	—	GH at 204 months	NS	Briggs et al. (case 1)
8	18	2	1.5 NS	Tumorectomy	—	GH at 60 months	NS	Briggs et al. (case 2)
9	14	3	6 NS	Tumorectomy	—	Death cause? At 24 months	NS	Briggs et al. (case 3)
10	13	+3.5/2 months	5 NS	Tumorectomy	—	GH at 19 months	Stroma lipofibroma sarcoma	Grigioni et al. (case I)
11	18	+15/2 months	15 NS	Radical mastectomy	Recurrence at 3 months, pulmonary metastasis at 9 months	Death at 10 months	Very aggressive	Grigioni et al. (case 2)
12	18	84	12 × 12 left, nipple discharge	Tumorectomy	2 recurrences at 2 months, 6 months	GH at 42 months	NS	Naryshkin and Redfield
13	12	NS	10 × 12.5 × 15 right, no menarche	Radical mastectomy	—	GH at 12 months	Negative ganglia	Gibbs et al.
14	17	NS	30 axillary adenopathy	Radical mastectomy	—	GH at 96 months	Ganglionic invasion	Long et al.
15	14	4	10 right	Subcutaneous mastectomy and axillary clearing out	Multirecurrences as soon as 1 month, pulmonary and ovarian metastases, radical mastectomy, metastasectomy and exeresis of recurrences, chemotherapy mono then polychemotherapy, cobaltherapy, surgical castration	Death at 14 months	Inflammatory reactive axillary ganglia	Hoover et al.
16	18	12	18 × 6 right, orange peel	Tumorectomy	3 recurrences at 4 months, 8 months, 19 months tumorectomy then radical mastectomy with radiotherapy, pulmonary metastases at 19 months	Death at 19 months	Almost sarcomatous tumour	Kenda
17	14	4	10 suspect left homolateral axiallty adenopathy	Subcutaneous mastectomy and axillary image radiotherapy, polychemotherapy	Metastases at 2 months (liver, bones, and lungs)	Death at 2 months	Necrosis and haemorrhage, normal epithelium, fibrosarcoma, >10 mitoses/10 HPF, periductular stromal hyper development, 8 reactive ganglia, 2 ganglia, purely stromal metastases	Ogun et al.
18	15	1 NS	Painful mass in left breast	Subcutaneous mastectomy	—	Death at 22 months	NS	Turalba et al.
19	15	4	16 × 6 cm	Enlarged tumorectomy, axillary clearing followed by mastectomy	—	GH at 100 months	Consistent with typical malignant pathology	Levique et al.
20	18	10	3 × 3 cm, right	Lumpectomy with wide local excision followed by simple mastectomy	Recurrence at 1.5 months following lumpectomy and 2 months following mastectomy	Death months following brain metastasis	Spindle cells with slight nuclear variation	Tiwari et al.
21	17	0.25	15 × 11 cm, right	Lumpectomy	—	GH at 2 month	Consistent with typical malignant pathology	Makar et al.

GH: good health; NS: not stated. Table adopted with permissions and additionas from Levêque et al. [14].

**Table 2:** Grading system for phyllodes tumors based on 2012 World Health Organization classification.

Histologic features	Histological type		
	Benign	Borderline	Malignant
Stromal cellularity	Mild	Moderate	Marked
Stromal atypia	Mild	Moderate	Marked
Mitosis (per 10 HPF)	<5	9-may	≥10
Stromal overgrowth	Absent	Absent or focal	Present
Tumor margin	Clear	Clear or infiltration	Infiltration

HPF: high-power field. Table adopted with permissions from Zhang and Kleer [34].
